# Efficient Exploitation of Multiple Novel Bacteriocins by Combination of Complete Genome and Peptidome

**DOI:** 10.3389/fmicb.2018.01567

**Published:** 2018-07-13

**Authors:** Lanhua Yi, Lingli Luo, Xin Lü

**Affiliations:** Department of Food Nutrition and Safety, College of Food Science and Engineering, Northwest A&F University, Yangling, China

**Keywords:** novel bacteriocins, genome, peptidome, cloning and expression, antibiotic-resistance

## Abstract

**Backgroud:** The growing emergence of antibiotic-resistant pathogens including the most dangerous superbugs requires quick discovery of novel antibiotics/biopreservatives for human health and food safety. Bacteriocins, a subgroup of antimicrobial peptides, have been considered as promising alternatives to antibiotics. Abundant novel bacteriocins are stored in genome sequences of lactic acid bacteria. However, discovery of novel bacteriocins still mainly relies on dubious traditional purification with low efficiency. Moreover, sequence alignment is invalid for novel bacteriocins which have no homology to known bacteriocins in databases. Therefore, an efficient, simple, universal, and time-saving method was needed to discover novel bacteriocins.

**Methods and Results:** Crude bacteriocins from both cell-related and culture supernatant of *Lactobacillus crustorum* MN047 fermentation were applied to LC-MS/MS for peptidome assay, by which 131 extracellular peptides or proteins were identified in the complete genome sequence of *L. crustorum* MN047. Further, the genes of suspected bacteriocins were verified by expressed in *Escherichia coli* BL21 (DE3) pLysS. Thereafter, eight novel bacteriocins and two nonribosomal antimicrobial peptides were identified to be broad-spectrum activity against both Gram-positive and Gram-negative bacteria, including some multidrug-resistant strains. Among them, BM1556 located within predicted bacteriocin gene cluster. The most active bacteriocin BM1122 had low MIC values of 13.7 mg/L against both *Staphylococcus aureus* ATCC29213 and *E. coli* ATCC25922. The BM1122 had bactericidal action mode by biofilm-destruction, pore-formation, and membrane permeability change.

**Conclusions:** The combination of complete genome and peptidome is a valid approach for quick discovery of novel bacteriocins without/with-low homology to known ones. This method will contribute to deep exploitation of novel bacteriocins in genome of bacteria submitted to GenBank.

## Introduction

Since the discovery of penicillin in 1929, antibiotics have played an indispensable role in human medicine and food production. Unfortunately, antimicrobial resistance is found in all microorganisms, whether bacteria, fungi, virus or parasites (Sabtu et al., 2015). Moreover, many bacteria are resistant to not only single but multiple antibiotics. In 2014, 480,000 new cases of multidrug-resistant tuberculosis (MDR-TB) were estimated by WHO. Among MDR-TB, 9.7% were extensively drug-resistant tuberculosis (XDR-TB), which was identified in 105 countries. *Klebsiella pneumoniae* resistant to carbapenems, which is usually the last line of available treatment, was reported in all WHO regions (Band et al., 2018). According to “global PPL” published by WHO in 2017, the most important resistant bacteria had resistance to carbapenem, 3rd generation cephalosporin, vancomycin, methicillin, clarithromycin, etc. Antibiotic resistance makes the existing drugs become increasingly ineffective or even invalid. Consequently, many common infections are becoming risky or untreatable, leading to longer illnesses and higher mortality, like returning to the pre-antibiotic era. The Centers for Disease Control and Prevention (CDC) estimated that antimicrobial-resistance had caused at least two million illnesses and 23,000 deaths each year in the United States alone (Leal et al., 2017), leading to health and economic burden up to $55 billion directly or indirectly. Undoubtedly, antibiotic resistance has become one of the biggest threats to global health in the twenty-first century (Arango-Argoty et al., 2018). However, the discovery of new antimicrobial drugs lags behind the emergence of superbugs. Recently, bacteriocins have attracted much attention because they are not only used as food preservatives but also looked as promising alternatives to antibiotics (Cotter et al., 2013). Bacteriocins are ribosomally synthesized antimicrobial peptides or proteins produced by bacteria with narrow or broad antibacterial spectrum (Cotter et al., 2005). Many bacteriocins are produced by lactic acid bacteria which are generally recognized as safe (GRAS) (Alvarez-Sieiro et al., 2016).

Although hundreds of bacteriocins have been discovered, only nisin has been widely used as food preservative in more than fifty countries. The limitation of bacteriocins as preservatives and antimicrobials in the market mainly derives from narrow-spectrum inhibitory activity and high cost of their commercial production (Fahim et al., 2016). Thus, exploitation of novel bacteriocins with broad-spectrum activity is vital for the development of antibiotic substitutes. Generally, novel bacteriocins are obtained and characterized through traditional purification process combining with mass spectrum (Stern et al., 2006). Okubo et al. (2012) and Ngoc Hieu et al. (2016) purified the proteins, and then applied them to LC-MS/MS to *de novo* sequencing. Kuyama et al. (2015) purified a human basic fetoprotein, which was subsequently identified by N/C-terminal sequencing. However, the traditional method is time-consuming, most importantly, the uncertainty of novelty makes the research easy to become meaningless. Further, more than one kind of bacteriocins can be produced by lactic acid bacteria, which adds the difficulty by traditional purification. In addition, processes of traditional purification vary with different samples as the component diversity.

Comparing with traditional purification, bacteriocin gene sequence alignment is much more simple, clear and, convenient (Porto et al., 2017). Moreover, another sequence search way of pattern-matching, such as profile Hidden Markov Models (profile-HMM) and regular expressions (REGEX) (Porto et al., 2017), had higher efficiency and higher precision. Lactic acid bacteria (LAB, e.g., *Lactococcus, Enterococcus, Oenococcus, Pediococcus, Streptococcus, Leuconostoc*, and *Lactobacillus* species) are the richest sources for bacteriocin production (Makarova et al., 2006). Many genome data of these LAB were stored in GenBank of NCBI. For example, only for *Lactobacillus*, complete or draft genome sequences of 173 species were submitted. Bacteriocin-coding genes can be identified according to their identity with known bacteriocins in databases. However, the novel bacteriocins, which are possible promising, will be overlooked by sequence alignment if they are not very similar to known genes (Tatusova et al., 2016). Therefore, an efficient, simple, universal and time-saving method was needed to discover more novel bacteriocins.

In the study of Dong et al. (2014), a LC-MS/MS-based *de novo* sequencing assisted database search was used to identified phosphopeptides, which simplified the whole analysis. However, the used database gathering abundant species limited specificity and accuracy. In this study, with experiments-based function confirmation, a combined method of complete genome of bacteriocin-producer itself and peptidome was used to specifically discover novel bacteriocins produced by probiotic *L. crustorum* MN047. The antimicrobial activities of multiple bacteriocins against pathogens were investigated, including against antibiotic-resistant isolates. Then, action mode of the most activity bacteriocin was preliminarily studied.

## Materials and methods

### Function annotation

Complete genome of *L. crustorum* MN047 was sequenced in our previous study (Yi et al., 2017). Function annotation was performed using local alignment with databases of NR (Non-Redundant Protein Database), Swiss-Prot, TrEMBL, COG (the Clusters of Orthologous Groups of proteins), KEGG (the Kyoto Encyclopedia of Genes and Genomes), and GO (Gene Ontology). Genes potentially related with bacteriocin were identified using BAGEL4 (http://bagel4.molgenrug.nl/) and antiSMASH (http://antismash.secondarymetabolites.org) (Liu et al., 2016).

### Peptidome

The *L. crustorum* MN047 was statically incubated in MRS medium (2 L) at 30°C for 60 h, after which cell suspension was divided into two equal parts to prepare crude bacteriocin samples. One was prepared by ammonium sulfate precipitation as previous study (Yi et al., 2016). The other was made by pH-mediated cell adsorption-desorption method (Yang et al., 1992). For the latter, adsorption and desorption pH value was 5.85 and 2.10, respectively. Briefly, cell suspension was heated at 70°C for 30 min to kill cells and inactivate enzymes. Then, pH of cell suspension was adjusted to 5.85 and slowly stirred at 4°C for 120 min. Cells were harvested by centrifugation and washed by citric acid-phosphate buffer (pH 5.85) for 3 times. Thereafter, pH of cell suspension was adjusted to 2.10. Supernatant was obtained by centrifugation and its pH value was adjusted to 7.0, namely, crude bacteriocin was acquired.

In order to identify potential bacteriocins produced by *L. crustorum* MN047, the two samples were respectively applied to LC-MS/MS, a Q Exactive mass spectrometer that was coupled to LC-20AD (Shimadzu, Japan). Samples were pretreated using Amicon Ultra centrifugal Filters (10 K, Millipore, USA). Filtered component was loaded onto a Trap column at 8 μL/min, followed by a LP-C18 analytical column (180 mm length × 76 μm i.d., 3 μm) at 300 nL/min under gradient elution. MS and MS/MS data were acquired and switched under DDA (data dependent acquisition) mode. Scan of mass-to-charge ratio was 350–1,800 m/z at resolution of 70,000 for MS and HCD (high-energy collisional dissociation) fragmentation was used at resolution of 17,500 for MS/MS. Raw files of MS/MS spectra were applied to Maxquant 1.5.2.8 (Cox and Mann, 2008) and MASCOT 2.2 (Koenig et al., 2008) against database of complete genome sequence of *L. crustorum* MN047, as well as databases of antimicrobial peptide APD3 (Wang et al., 2016) (http://aps.unmc.edu/AP/) and CAMP (Waghu et al., 2016) (http://www.camp3.bicnirrh.res.in/). Function annotation of suspicious bacteriocins was conducted by InterPro (http://www.ebi.ac.uk/interpro/) (Jones et al., 2014).

### Heterologous expression of bacteriocins

*Escherichia coli* expression system was used to obtain each one of suspicious bacteriocins. The pET-30a (Novagen, Germany) and *E. coli* BL21 (DE3) pLysS (Trans, China) were used as expression vector and expression host, respectively. Genome of *L. crustorum* MN047 was extracted and used as template to amplify genes of 8 hypothetical bacteriocins with 8 pairs of primers shown in Table 1. All PCR reactions ran for 35 cycles under corresponding annealing temperature of each primer pair and PCR products were purified using Gel Extraction Kit (OMEGA, USA). After which, PCR products were inserted into pET-30a by digested with *FlyCut*^TM^ *Nde* I and *FlyCut*^TM^ *Hind* III and ligated with T4 Ligase (all from Trans, China). Specifically, there were no encoding genes on genome for nonribosomal antimicrobial peptide EP-20 and GP-19, so their encoding genes were directly synthesized (Sangon, China) (Table 1) according to amino acid sequence. These recombinant plasmids with specific bacteriocin gene were separately transformed into *E. coli* BL21 (DE3) pLysS competent cells by heat shock at 42°C. The transformants with correct insertion of bacteriocin genes were confirmed by PCR amplification and sequencing.

**Table 1:** Oligonucleotide primers used in PCR amplification and synthesized genes in this study.

**Primers**	**Sequence (5^′^ → 3^′^)**	**Restriction site**
BM173F	GGGAATTCCATATGTCACAAAATACACATAAAGG	*Nde* I
BM173R	CCCAAGCTTTTATTTAGATTCTTTAGTATCACGG	*Hind* III
BM797F	GGGAATTCCATATGAGATATAAAGTTACTTTAG	*Nde* I
BM797R	CCCAAGCTTCTATGCACTCATTTTCAATAAT	*Hind* III
BM1029F	GGGAATTCCATATGGATAAACCAAATATTGCTGAAAT	*Nde* I
BM1029R	CCCAAGCTTTTATTGATGCAATTTGATATATTCTTGA	*Hind* III
BM1122F	GGGAATTCCATATGGCAAATAAAGCTGAACTTATTG	*Nde* I
BM1122R	CCCAAGCTTTTATTTAACTGAGTCCTTCAAAGCCTTA	*Hind* III
BM1556F	GGGAATTCCATATGAAAAGAATATTGTTAAAGTC	*Nde* I
BM1556R	CCCAAGCTTTTAATGCCAGCGTTGATCAAT	*Hind* III
BM1829F	GGGAATTCCATATGGCAGAAGTAGATCCATCAAAGAT	*Nde* I
BM1829R	CCCAAGCTTTTATTTTTTAAGGTTTTTTTCGACAAAATCACG	*Hind* III
BMP11F	GGGAATTCCATATGAGTATAAATAAGCAAAAAATTTCG	*Nde* I
BMP11R	CCCAAGCTTTTATTTTCCCTTTTTAATGGTCTTAATGA	*Hind* III
BMP32F	GGGAATTCCATATGACAGTCACAGATCCGCGTAGTCCG	*Nde* I
BMP32R	CCCAAGCTTTCAAGCGATCATCGCGCCACTACTCGTT	*Hind* III
EP-20	GGGAATTCCATATGGAAGGTCCGGTTGGTCTGGCAGA TCCGGATGGTCCGGCAAGCGCACCGCTGGGTGCACCG TAA*AAGCTT*GGG	*Nde* I, *Hind* III
GP-19	GGGAATTCCATATGGGTCCGGTTGGTCTGCTGAGCAG CCCGGGTAGCCTGCCGCCGGTTGGTGGTGCACCGTAA *AAGCTT*GGG	*Nde* I, *Hind* III

*F, forward primer; R, reverse primer; the underlined sequence is restriction enzyme site*.

*Escherichia coli* BL21(DE3) pLysS carrying recombinant vector was incubated in 100 mL LB broth containing kanamycin to an OD_600_ of 0.6 at 37°C (shaking at 150 rpm), and then induced by isopropyl β-D-thiogalactoside (IPTG) over night at 25°C (shaking at 180 rpm). Cells were harvested by centrifugation (4,000 × g, 4°C, 15 min), washed by PBS (pH 7.2) for three times, and resuspended in 20 mM Tris-HCl (pH 6.68). Cells were repeated 5 freeze-thaw cycles to disrupt cells and heated at 80°C to kill cells and inactivate enzymes. The supernatants (1 mL) were collected by centrifugation (16,000 × g, 4°C, 15 min) and applied to antimicrobial activity test by agar well diffusion method as previous study (Lü et al., 2014) using *S. aureus* ATCC29213 as indicator. The concentrations of kanamycin and IPTG of each bacteriocin were optimized until an obvious antibacterial activity was gotten. The same treatment of each bacteriocin transformant without inducement was used as control in antimicrobial activity test. One arbitrary unit (AU) was defined as the reciprocal of the highest dilution of the bacteriocin showing a visible clear zone (Jabrane et al., 2002).

### Antimicrobial spectrum

Some Gram-positive and Gram-negative foodborne pathogenic bacteria including multidrug-resistant strains (Table 2) were used to investigate the inhibitory spectrum of 8 bacteriocins and 2 nonribosomal antimicrobial peptides. Supernatant of cell disruption was used and antimicrobial activity toward all indicators was measured by the agar well diffusion assay.

**Table 2:** The inhibitory spectrum of 8 novel bacteriocins and 2 nonribosomal antimicrobial peptides.

**Indicator strains**	**Diameter of inhibition zone (mm)**									
	**BM173**	**BM797**	**BM1029**	**BM1122**	**BM1556**	**BM1829**	**BMP11**	**BMP32**	**EP-20**	**GP-19**
**GRAM-POSITIVE**										
*Staphylococcus aureus* ATCC29213	21.4 ± 0.3	20.0 ± 0.7	20.8 ± 0.2	23.1 ± 0.8	15.9 ± 0.1	18.8 ± 0.2	22.0 ± 0.6	19.7 ± 0.6	15.3 ± 0.2	19.2 ± 0.7
*S. aureus* ATCC25923	21.6 ± 0.4	19.9 ± 0.1	20.9 ± 0.4	24.4 ± 0.7	16.0 ± 1.1	18.8 ± 0.1	22.8 ± 0.4	19.0 ± 0.1	17.4 ± 0.6	19.1 ± 0.5
*Enterococcus faecalis* ATCC29212	12.6 ± 1.4	0	16.0 ± 0.2	12.9 ± 0.2	0	11.0 ± 0.3	13.6 ± 0.5	15.2 ± 0.4	10.2 ± 1.1	10.3 ± 0.3
*Listeria monocytogenes* CMCC54004	19.5 ± 0.5	17.5 ± 0.4	19.1 ± 1.0	21.7 ± 0.8	12.8 ± 0.5	17.9 ± 0.3	21.8 ± 0.6	16.7 ± 0.5	12.1 ± 1.6	17.2 ± 0.9
Antibiotic-resistant *S. aureus* 1^a^	12.7 ± 0.6	17.5 ± 0.2	0	13.2 ± 0.7	0	0	17.3 ± 0.6	0	0	0
Antibiotic-resistant *S. aureus* 2^b^	0	17.6 ± 0.6	0	0	0	0	9.8 ± 0.5	0	0	0
Antibiotic-resistant *S. aureus* 3^c^	11.2 ± 0.5	18.1 ± 1.7	0	10.6 ± 0.7	0	0	13.7 ± 0.5	0	0	0
Antibiotic-resistant *S. aureus* 4^d^	0	15.6 ± 0.7	0	11.6 ± 1.0	0	0	13.1 ± 0.7	0	0	0
Antibiotic-resistant *S. aureus* 5^e^	0	13.0 ± 0.8	0	0	0	0	12.0 ± 1.2	0	0	0
**GRAM-NEGATIVE**										
*Escherichia coli* ATCC25922	20.2 ± 0.4	14.4 ± 0.7	19.1 ± 0.5	20.9 ± 0.4	13.0 ± 0.6	17.0 ± 1.0	20.7 ± 0.9	17.4 ± 0.5	12.3 ± 0.7	17.0 ± 1.6
*Salmonella* CMCC 50071	26.7 ± 0.7	14.6 ± 0.3	22.7 ± 1.5	27.3 ± 1.5	17.6 ± 1.4	17.7 ± 0.6	23.6 ± 1.3	20.6 ± 0.8	12.1 ± 0.4	18.7 ± 0.5
*Cronobacter sakazakii* ATCC29544	18.7 ± 1.0	10.8 ± 0.4	19.3 ± 0.4	22.7 ± 0.7	16.1 ± 0.3	19.4 ± 0.3	21.5 ± 0.1	17.6 ± 0.5	15.0 ± 1.1	18.0 ± 0.7
Antibiotic-resistant *Salmonella* 36T^f^	0	0	0	0	0	0	0	0	0	0
Antibiotic-resistant *Salmonella* 87T4^g^	0	0	0	0	0	0	0	0	0	0
Antibiotic-resistant *Salmonella* 557D^h^	19.7 ± 0.7	9.6 ± 0.3	17.7 ± 1.1	22.0 ± 0.9	13.1 ± 0.4	13.9 ± 0.6	21.5 ± 0.4	18.6 ± 1.7	12.0 ± 1.1	15.6 ± 1.4
Antibiotic-resistant *Salmonella* 798D^i^	0	0	0	0	0	0	0	0	0	0
Antibiotic-resistant *Salmonella* 1006D^j^	0	0	0	0	0	0	0	0	0	0
Antibiotic-resistant *C. sakazakii* 6–12 (1)^k^	19.5 ± 0.3	10.4 ± 1.0	20.5 ± 0.4	22.3 ± 0.5	15.4 ± 0.9	18.0 ± 0.3	22.9 ± 0.9	18.4 ± 0.3	14.6 ± 0.3	16.9 ± 1.0
Antibiotic-resistant *C. sakazakii* 6–12 (2)^l^	20.3 ± 0.6	10.8 ± 0.5	17.8 ± 0.6	21.9 ± 0.9	15.7 ± 1.2	17.4 ± 0.3	22.7 ± 0.4	19.3 ± 1.0	14.9 ± 0.9	18.4 ± 0.4
Antibiotic-resistant *C. sakazakii* 11–18 (2)^m^	17.9 ± 0.7	10.9 ± 0.4	20.0 ± 0.2	21.5 ± 0.4	16.0 ± 1.2	18.3 ± 0.5	21.6 ± 1.3	18.5 ± 0.4	15.1 ± 0.2	17.4 ± 0.4
Antibiotic-resistant *C. sakazakii* 14–18 (2)^n^	22.3 ± 0.7	9.9 ± 0.3	20.4 ± 0.7	23.5 ± 0.9	16.8 ± 0.6	18.3 ± 0.2	23.2 ± 0.9	21.2 ± 0.6	15.7 ± 0.6	19.4 ± 1.2
Antibiotic-resistant *C. sakazakii* 18–15 (2)^o^	20.3 ± 0.6	9.4 ± 0.3	20.1 ± 0.8	21.8 ± 0.2	15.0 ± 0.2	17.8 ± 0.4	22.4 ± 0.2	17.5 ± 0.3	18.1 ± 0.8	17.7 ± 0.1

a Resistant to cephalothin, ciprofloxacin, clarithromycin, cefuroxime, cefoxitin, gentamicin, levofloxacin, tobramycin, ofloxacin, oxacillin, piperacillin, vancomycin.

b Resistant to ciprofloxacin, cefoxitin, gentamicin, levofloxacin, minocycline, tobramycin, ofloxacin, penicillin, piperacillin, vancomycin.

c Resistant to ciprofloxacin, cefoxitin, gentamicin, levofloxacin, minocycline, tobramycin, ofloxacin, vancomycin.

d Resistant to ciprofloxacin, cefoxitin, gentamicin, levofloxacin, minocycline, tobramycin, ofloxacin, vancomycin.

e Resistant to ciprofloxacin, cefoxitin, gentamicin, levofloxacin, minocycline, tobramycin, ofloxacin, oxacillin, vancomycin.

f Resistant to ampicillin, amoxicillin, amikacin, chloramphenicol, ciprofloxacin, cefoperazone, ceftriaxone, gatifloxacin, gentamicin, kanamycin, levofloxacin, nalidixic acid, trimethoprim-sulfamethoxazole, tetracycline.

g Resistant to ampicillin, amoxicillin, amikacin, chloramphenicol, ciprofloxacin, cephalothin, cefoperazone, ceftriaxone, gatifloxacin, gentamicin, kanamycin, levofloxacin, nalidixic acid, streptomycin, trimethoprim-sulfamethoxazole, tetracycline.

h Resistant to ampicillin, amoxicillin, amikacin, chloramphenicol, cefoxitin, gatifloxacin, gentamicin, kanamycin, levofloxacin, nalidixic acid, trimethoprim-sulfamethoxazole, tetracycline.

i Resistant to ampicillin, amoxicillin, amikacin, chloramphenicol, ciprofloxacin, cephalothin, ceftriaxone, cefoxitin, gatifloxacin, gentamicin, kanamycin, levofloxacin, nalidixic acid, streptomycin, trimethoprim-sulfamethoxazole, tetracycline.

j Ampicillin, amoxicillin, chloramphenicol, gentamicin, kanamycin, nalidixic acid, trimethoprim-sulfamethoxazole, tetracycline.

k Cefoxitin, rifampin.

l Chloramphenicol, rifampin, tetracycline.

m Ciprofloxacin, rifampin, streptomycin.

n Ciprofloxacin, rifampin, streptomycin.

o *Ampicillin, amoxicillin, chloramphenicol, nalidixic acid, rifampin, streptomycin, tetracycline*.

### Purification of BM1122

Crude bacteriocin BM1122 was dialyzed in a dialysis tube with MW 8,000. Then, the sample within tube passed through an Amicon Ultra centrifugal Filter (10 K). The permeate was concentrated and purified on an AKTA system (AKTA Purifier 100, GE, Sweden) equipped with an UV detector and an automatic collector. Sample was loaded on a HiTrap Q FF anion-exchange column at 1.5 mL/min. Citric acid-phosphate buffer (pH 6.0) was used as equilibrium buffer. Sample was gradiently eluted with 1 M NaCl in equilibrium buffer. Then, the active fraction was purified by analytical RP-HPLC (Waters 1525, USA) equipped with an Agilent ZORBAX 300SB-C18 column (250 × 4.6 mm, 5 μm). The mobile phase A (80% H_2_O, 20% acetonitrile and 0.05% TFA) and mobile phase B (100% acetonitrile) were used in gradient elution. The purified BM1122 was digested by trypsin (37°C, 16 h), and then desalted by a manual Pierce C18 Tips (USA, Thermo Scientific). Subsequently, the sample was applied to LC-MS/MS as above to identify the BM1122.

### MIC value

The BM1122 from RP-HPLC was concentrated and applied to measurement of MIC value as described method (Bhattacharyya et al., 2017) in 96 well plate (Costar, Corning, USA). *S. aureus* ATCC 29213 and *E. coli* ATCC 25922 were used as indicators. Specifically, indicators were cultured at 37°C to log-phase and diluted using fresh LB broth to about 10^6^ CFU/mL. The initial concentration of BM1122 was adjusted to 2 g/L using sterile PBS buffer, and then it was double diluted to 2^−1^, 2^−2^, 2^−3^, 2^−4^, 2^−5^, 2^−6^, 2^−7^, 2^−8^, 2^−9^, and 2^−10^. Ten microliter of each BM1122 dilution and 90 μL indicator dilution were mixed in wells, and then incubated at 37°C for 24 h. MIC was defined as the lowest concentration at which no growth of indicator was observed. Meanwhile, protein concentration of each diluted bacteriocin was measured again by BCA assay in case of deviations of concentrations during serial dilution and BSA was used as standard.

### Time-kill curve

Foodborne pathogen *L. monocytogenes* CMCC54004 and *C. sakazakii* ATCC29544 were used as indicators. Indicators were cultured at 37°C to logarithmic-phase (10^6^–10^8^ CFU/mL), and then sample BM1122 was added to a final concentration of 4 × MIC. Indicators were continuously incubated at 37°C, and 0.5 mL bacterial suspensions were taken immediately at appointed times (0.5, 1, 1.5, and 2 h). Cell suspensions were 10-fold serially diluted using sterile PBS buffer (pH 7.2). Cells of two appropriate serial dilutions were spread on plates of LB medium with triplicate. Bacterial colonies were counted after cultivation at 37°C for 24 h. Indicators without bacteriocin were used as controls.

### Scanning electron microscope and transmission electron microscope

Electron microscopes were used to visualize action mode of BM1122. Foodborne pathogen *L. monocytogenes* CMCC54004 and *C. sakazakii* ATCC29544 were used as indicators. Exponential-phase indicators were treated by a concentration of 2 × MIC bacteriocin at 37°C for 0.5 and 2 h, respectively. After washing, fixation and dehydration as described method (Yi et al., 2016), cells were dried by CO_2_ and coated with gold. Ultrastructure of indicators was observed using a high resolution Nova NanoSEM 450 scanning electron microscope (SEM) (FEI, USA).

Pretreatment of indicators for transmission electron microscope (TEM) was the same with that for scanning electron microscope. After post-fixed by osmic acid, cells were dehydrated using alcohol and permeated using white resin. Embedding was performed by roasting at 55°C for 48 h. Seventy nanometer thin sections were prepared on copper grids and stained with lead citrate and uranyl acetate. Ultrastructure observation was conducted on a Tecnai G2 Spirit Bio-Twin TEM (FEI, USA).

### Data availability

All sequence data that support the findings of this study have been deposited in GenBank under accession numbers: CP017996 (chromosome), CP017997 (plasmid MN047p1), and CP017998 (plasmid MN047p2).

## Results

### Function annotation

**Figure 1:**
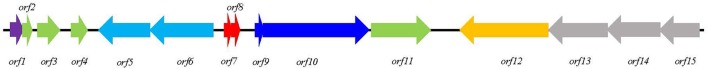
Schematic overview of the bacteriocin gene cluster predicted in *L. crustorum* MN047 (drawn in scale). Orf1, hypothetical protein (transcriptional regulator, 47%); orf2, hypothetical protein (IS5/IS1182 family transposase, 94%); orf3, hypothetical protein (IS5/IS1182 family transposase, 55%); orf4, hypothetical protein (IS5/IS1182 family transposase, 63%); orf5, hypothetical protein (ABC transporter permease, 46%); orf6, bacteriocin ABC transporter ATP-binding protein; orf7, hypothetical protein; orf8, hypothetical protein; orf9, hypothetical protein (bacteriocin ABC transporter, 58%); orf10, bacteriocin ABC transporter; orf11, IS30 family transposase; orf12, Magnesium transporter; orf13, RluA family pseudouridine synthase; orf14, NAD kinase; orf15, GTP pyrophosphokinase. Putative bacteriocin structure genes (red), ABC transporter genes (blue), transport-related genes (light blue), regulatory related genes (green), transcriptional regulator (purple), metal resistance gene (orange), and other genes (gray).

*Lactobacillus crustorum* MN047, firstly isolated from koumiss, which could produce at least three bacteriocins in our previous study (Yi et al., 2016). Complete genome sequence was investigated to analyze genes related to antimicrobial activity. There were 2218 protein coding genes after prediction of its complete genome sequence. A total of 2,176 genes were assigned a putative function by elaborate annotation based on local alignment using six databases. A gene cluster of bacteriocin (locus_tag: BI355_1567-BI355_1581) (Figure 1) was identified using secondary metabolite-specific database antiSMASH. Among the 15 putative *orf* s, homologous proteins of the proteins encoded by *orf* 6, *orf* 9, and *orf* 10 were widely found in other bacteriocin biosynthetic gene clusters (Smokvina et al., 2013; Toh et al., 2013). The structure of bacteriocin gene cluster predicted in *L. crustorum* MN047 was similar to that of multipeptide leaderless bacteriocin family (Ovchinnikov et al., 2016), in which a metal resistance protein gene was included beside bacteriocin export/regulation related genes. Bacteriocin immunity gene was not included in the predicted bacteriocin gene cluster, which was a common feature among leaderless bacteriocins (Alvarez-Sieiro et al., 2016). One or both proteins from *orf* 7 and *orf* 8 were putative bacteriocin structural gene, which should be verified further. Although lack of genes encoding immunity protein in the bacteriocin gene cluster, four genes of bacteriocin immunity protein (locus_tag: BI355_0201, BI355_0202, BI355_2153, and BI355_2161) were found on chromosome far from the bacteriocin gene cluster. The multiple bacteriocin immunity proteins may be related to the multiple bacteriocins produced by *L. crustorum* MN047. In addition, a CvpA family protein (colicin V production protein, locus_tag: BI355_0565) was located on chromosome which might involve in bacteriocin biosynthesis.

However, no peptide or protein was annotated to be bacteriocin by database BAGEL3 and antiSMASH that no one had identity more than 50% with known bacteriocins in databases. Also, no encoding gene of bacteriocin BMA identified in our previous study (Yi et al., 2016) was found. Therefore, the BMA might be a nonribosomal antimicrobial peptide. Bacitracin is a nonribosomally synthesized peptide, two bacitracin export ATP-binding proteins (locus_tag: BI355_1593 and BI355_2140) were located on the chromosome of *L. crustorum* MN047. Moreover, an ABC transporter ATP-binding protein (locus_tag: BI355_1814) and four ABC transporter permeases (locus_tag: BI355_1140, BI355_1815, BI355_1888, and BI355_2139) of ABC-type antimicrobial peptide transport system were contained. They could be responsible for the transportation of nonribosomally synthesized peptides produced by *L. crustorum* MN047.

### LC-MS/MS

It is an obvious contradiction that multiple bacteriocins were found according to traditional purification process in our previous study while no related genes were annotated in the complete genome. There is only one possible reason that bacteriocins produced by *L. crustorum* MN047 are novel without any record in databases. Bacteriocins are extracellular antimicrobial peptides or proteins existed in both culture supernatant and cell associated form (Dündar et al., 2015). Ammonium sulfate precipitation can concentrate bacteriocins in culture supernatant. Moreover, pH-mediated cell adsorption-desorption method is good for cell associated bacteriocins. In this study, two methods above were used simultaneously to prepare crude bacteriocins.

**Figure 2:**
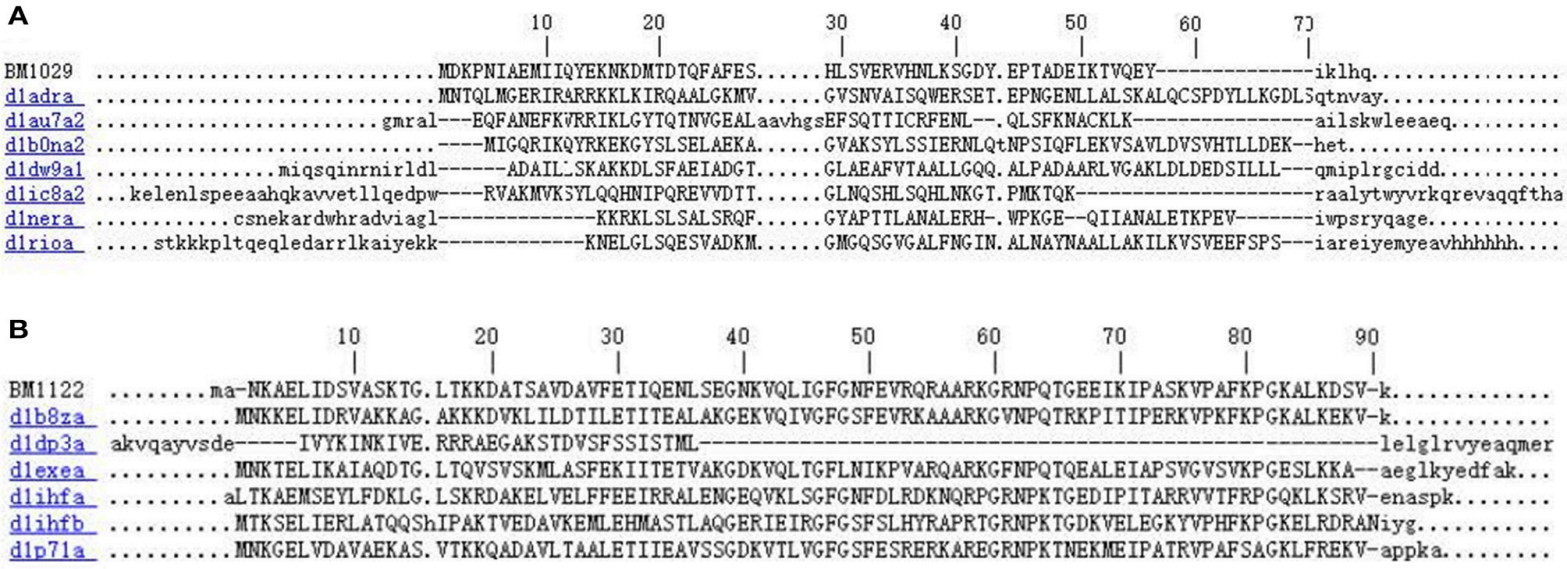
Sequence alignments of BM1029 **(A)** and BM1122 **(B)** with DNA-binding domain subfamily proteins by InterPro.

MS/MS data were matched to complete genome sequence of *L. crustorum* MN047, by which 66 and 170 peptide sequences were identified from ammonium sulfate precipitation sample and pH-mediated cell adsorption-desorption sample, respectively. They were fragments of 131 extracellular peptides or proteins of *L. crustorum* MN047 on chromosome or plasmid. The identified peptides with molecular mass < 10,000 and indefinite function annotation were focused. Finally, eight peptides were selected as suspicious bacteriocins (Table 3). Among them, the BM1556 was one of the putative structural genes of bacteriocin gene cluster above. The BM1029 was predicted to be putative λ repressor-like DNA-binding protein by InterPro according to structure similarity despite of a low sequence identity (Figure 2A). The BM1122 was annotated to be DNA-binding protein with a high sequence identity (Figure 2B). For the eight peptides, BM797 was from crude bacteriocin of ammonium sulfate precipitation, others were from pH-mediated cell adsorption-desorption sample. On the other hand, two nonribosomal antimicrobial peptides (EP-20 and GP-19) were also identified from the sample of ammonium sulfate precipitation after sequence alignment search in databases of APD3 and CAMP. The two antimicrobial peptides were previously found in symbiotic bacteria *Xenorhabdus budapestensis* NMC-10 (Xiao et al., 2012). In this study, a combined method of complete genome and peptidome was used to identify bacteriocins, in which the bacteriocins and nonribosomal antimicrobial peptides were not purified. Namely, the samples used in peptidome analysis also contained a mass of undesirable proteins or peptides from metabolites of *L. crustorum* MN047 and MRS medium (MRS medium contained peptone, beef extract and yeast extract). Therefore, the native masses of bacteriocins and nonribosomal antimicrobial peptides were not displayable by MS analysis as their very low abundance. All MS/MS data of bacteriocins and nonribosomal antimicrobial peptides were showed in Figure S1.

**Table 3:** Information statistics of targeted suspicious bacteriocins.

**NO**.	**Name^a^**	**Sequences**	**Function annotation^b^**
1	BM173	MSQNTHKGMTGHRRPVNQKNGAEKRAKTQAVLDFLRSRDTKESK	hypothetical protein
2	BM797	MRYKVTLDTKQQLFTVFDKKNTRVSACGKSIEEAMNKLLKMSA	hypothetical protein
3	BM1029	MDKPNIAEMIIQYEKNKDMTDTQFAFESHLSVERVHNLKSGDYEPTADEIKTVQEYIKLHQ	putative λ repressor-like DNA-binding protein
4	BM1122	MANKAELIDSVASKTGLTKKDATSAVDAVFETIQENLSEGNKVQLIGFGNFEVRQRAARKGRNPQTGEEIKIPASKVPAFKPGKALKDSVK	DNA-binding protein
5	BM1556	MKRILLKSDRTLDDSELAKVIGGGFFEGIGRWIDQRWH	putative bacteriocin
6	BM1829	MAEVDPSKMADAAIAKEPEVLNLKMSEAFDWSDDDTVVRDAIWDYFMENNNHDTVKTEEAEKPFLDMKDEEVRDFVEKNLKK	hypothetical protein
7	BMP11	MSINKQKISRNKVLNLLTLFQLLISLYQVIKTIKKGK	putative transmembrane protein
8	BMP32	MTVTDPRSPLTTWIFFCSKKTTPPLKGAVVMPNSGLSRHLHYLRLSSRCLSNSRNTPTSSGAMIA	hypothetical protein

a BM is the abbreviation of bacteriocin MN047.

b Predicted by InterPro (http://www.ebi.ac.uk/interpro/)

### Heterologous production and functional expression of bacteriocins

**Figure 3:**
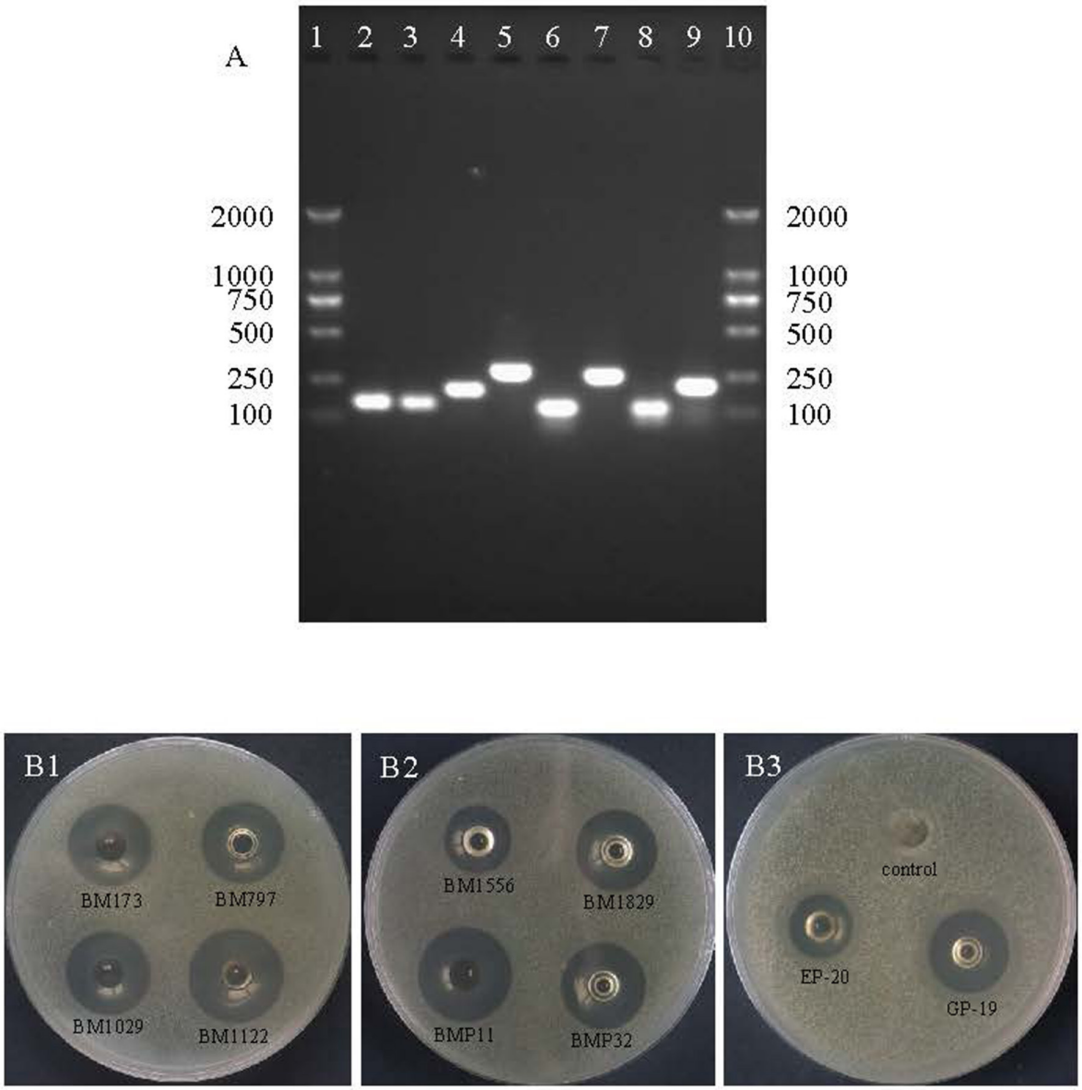
DNA agarose gel electrophoresis of PCR products of 8 hypothetical bacteriocins and inhibition zones against *S. aureus* ATCC29213. **(A)** Lane 1 and 10 are DNA marker (Novagen); lane 2 is gene of BM173; lane 3 is gene of BM797; lane 4 is gene of BM1029; lane 5 is gene of BM1122; lane 6 is gene of BM1556; lane 7 is gene of BM1829; lane 8 is gene of BMP11; lane 9 is gene of BMP32. **(B1)** Inhibition zones of BM173, BM797, BM1029, and BM1122; **(B2)** inhibition zones of BM1556, BM1829, BMP11, and BMP32; **(B3)** inhibition zones of EP-20, GP-19 and control without induction.

Non-fusion heterologous expression was used to verify these identified bacteriocin encoding genes. After PCR amplification using genome DNA of *L. crustorum* MN047 as template, genes of 8 hypothetical bacteriocins were all amplified with single band (Figure 3A). *E. coli* BL21 (DE3) pLysS was used as expression host to reduce the toxicity of bacteriocins or nonribosomal antimicrobial peptides under background expression.

After optimization of kanamycin and IPTG concentration (Table S1), the production and functional expression of bacteriocins were confirmed by antimicrobial activity test using agar well diffusion method. Consequently, the 8 hypothetical bacteriocins and 2 nonribosomal antimicrobial peptides all showed antimicrobial activity compared with control as shown in Figure 3B. The nonribosomal antimicrobial peptide EP-20 had the weakest antibacterial activity (320 AU/mL), the activity of bacteriocin BM797, BM1556, BM1829, and the other nonribosomal antimicrobial peptide GP-19 was 2-fold higher. Other bacteriocins BM173, BM1029, BM1122, BMP11, and BMP32 were 4-fold higher.

### Antimicrobial spectrum

Antimicrobial spectrum of 8 bacteriocins and 2 nonribosomal antimicrobial peptides was measured as shown in Table 2. All of which had broad-spectrum antimicrobial activity against both Gram-positive and Gram-negative bacteria. These peptides had no remarkable preference between Gram-positive and Gram-negative wild bacteria except for BM797 that the BM797 seemed to be more effective toward Gram-positive strains. The approximate order of inhibitory competence was BM1122 > BMP11 > BM173 > BM1029 > BM797 > BMP32 > GP-19 > BM1829 > BM1556 > EP-20. Moreover, they also showed antibacterial activity against multidrug-resistant strains. Strains of multidrug-resistant *S. aureus* used in this study were formidable with resistance to different kinds of antibiotics containing multiple lethal modes. The BM797 was specifically more powerful toward multidrug-resistant *S. aureus* than other bacteriocins. The 8 bacteriocins and 2 nonribosomal antimicrobial peptides revealed same antibacterial activity against strains of standard and resistant *Cronobacter sakazakii*. Among these bacteriocins and nonribosomal antimicrobial peptides, BM1122 exhibited superior activity, which was further studied.

### Purification of BM1122

**Figure 4:**
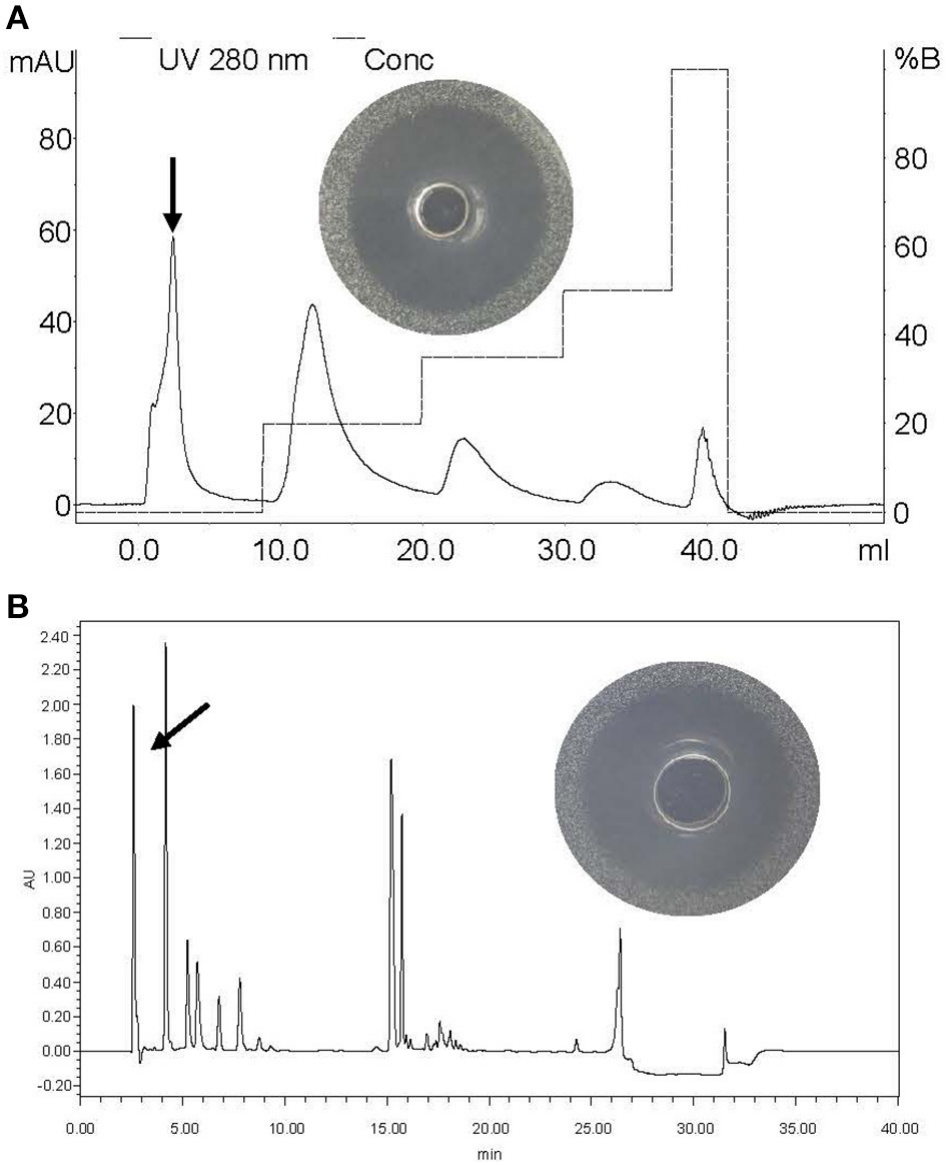
Purification of BM1122 by chromatographic columns. **(A)** Anion-exchange column and inhibition zone of arrowed peak. **(B)** RP-HPLC and inhibition zone of arrowed peak. The y-axis in **(B)** is the absorbance value (arbitrary units, AU) under 215 nm. Solid line is 280 nm, dotted line is conc.

**Figure 5:**
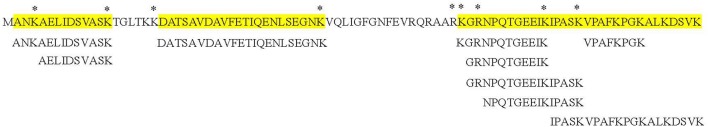
Covered residues of BM1122 by LC-MS/MS (highlight in yellow). * is the digestion sites of trypsin.

Crude BM1122 was firstly dialyzed, after which the sample was purified by an anion-exchange column as shown in Figure 4A. Five isolated peaks were obtained for BM1122 and the active peak (arrow in Figure 4A) was collected and applied to RP-HPLC. Subsequently, nine clear peaks were gotten (Figure 4B). The summit of active peak (arrow in Figure 4B) was collected. BM1122 was digested by trypsin which acted on arginine (R) or lysine (K). After analysis by LC-MS/MS, 9 fragments of BM1122 (Figure S2) were identified with a coverage of 73.63%. Sequences of both ends and middle were covered (Figure 5), which indicated that the active BM1122 was the core bacteriocin without any remove of N/C-terminal. The unidentified fragments of TGLTK and VQLIGFGNFEVR might attribute to loss during filtration and elution using manual C18 Tips to desalt after digestion.

### MIC value

*Staphylococcus aureus* ATCC 29213 and *E. coli* ATCC 25922, a representative of Gram-positive and Gram-negative bacteria, respectively, were used as indicators. As a result, the MIC value of BM1122 against both indicators was 13.7 mg/L.

### Action modes of BM1122

**Figure 6:**
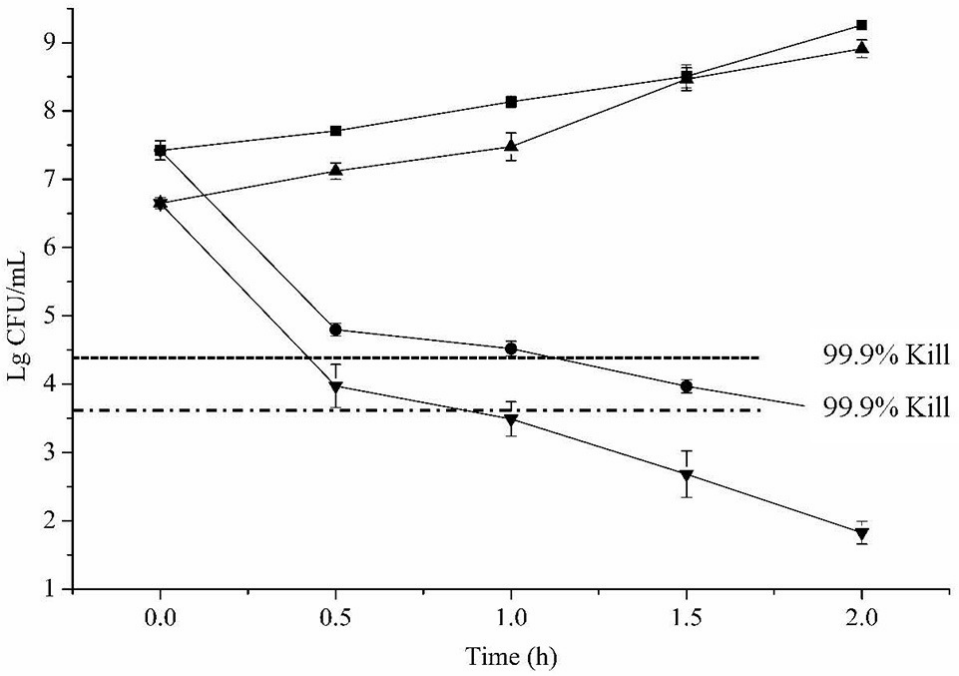
Time-kill cures. ■ is *L. monocytogenes* for control, • is *L. monocytogenes* treated by BM1122 at 4 × MIC, ▴ is *C. sakazakii* for control, ▾ is *C. sakazakii* treated by BM1122 at 4 × MIC.—- — — – is 99.9% (3log_10_ CFU/mL) kill of *L. monocytogenes* by bacteriocin;—- - — - – is 99.9% kill of *C. sakazakii* by bacteriocin.

Foodborne pathogen *L. monocytogenes* CMCC54004 and *C. sakazakii* ATCC29544 were used as indicators to investigate action mode of BM1122. Treatment with BM1122 for 1 h resulted in 2.9 log_10_ and 3.16 log_10_ reduction for *L. monocytogenes* and *C. sakazakii* (Figure 6), respectively. Viable cells further decreased with time, then all treatment caused more than 3 log_10_ (99.9%) reduction. Therefore, bacteriocin BM1122 was bactericidal (Kalia et al., 2009) at concentration of 4 × MIC.

**Figure 7:**
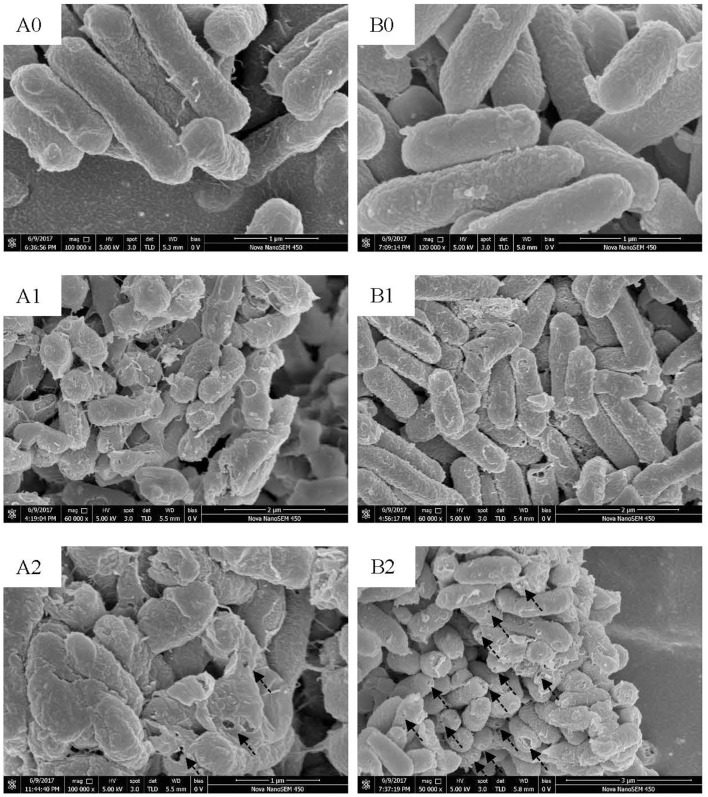
SEM images of *L. monocytogenes* [control **(A0)**; treated by BM1122 for 0.5 h **(A1)**; treated by BM1122 for 2 h **(A2)**] and *C. sakazakii* [control **(B0)**; treated by BM1122 for 0.5 h **(B1)**; treated by BM1122 for 2 h **(B2)**].

**Figure 8:**
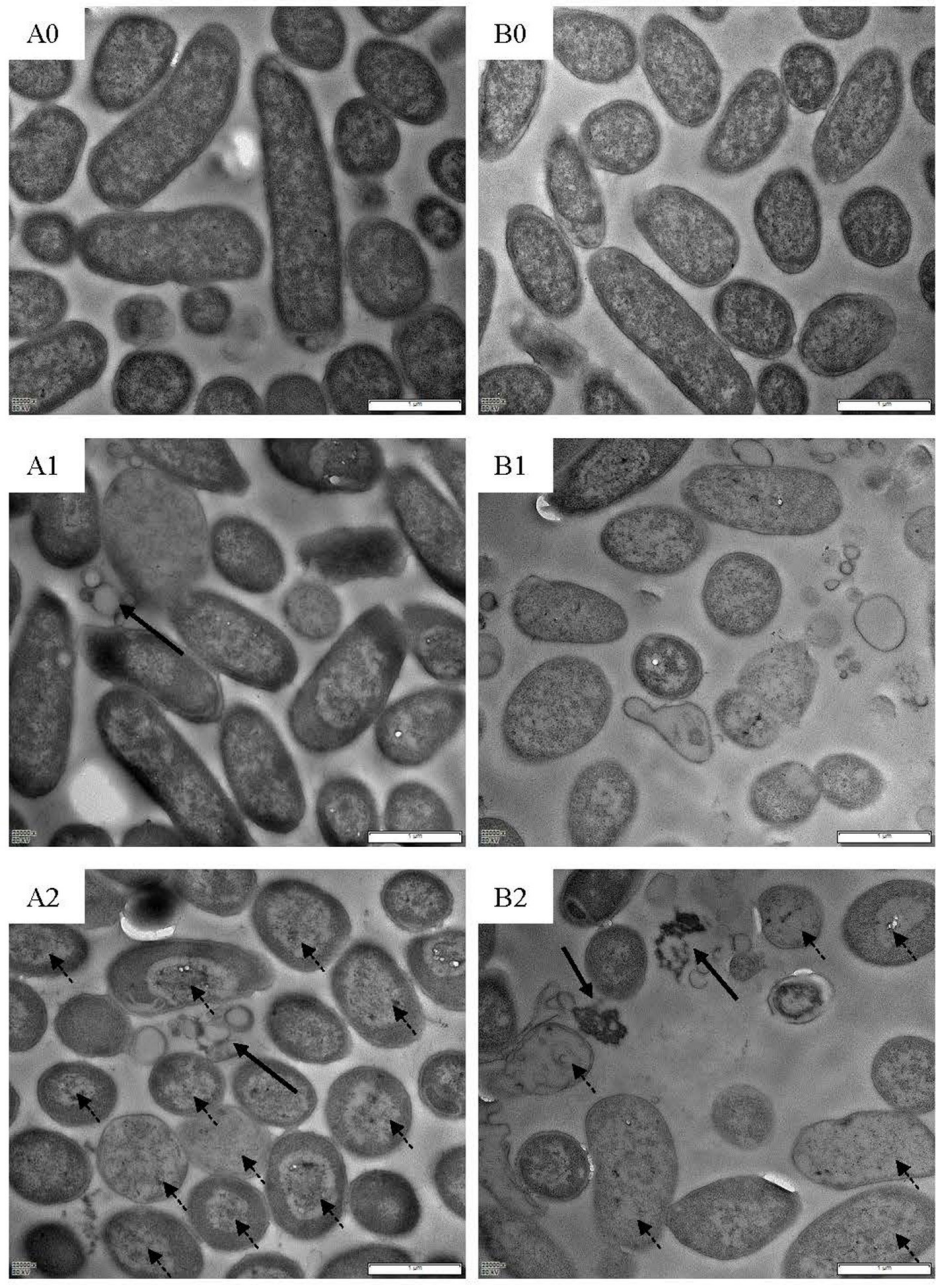
TEM images of *L. monocytogenes* [control **(A0)**; treated by BM1122 for 0.5 h **(A1)**; treated by BM1122 for 2 h **(A2)**] and *C. sakazakii* [control **(B0)**; treated by BM1122 for 0.5 h **(B1)**; treated by BM1122 for 2 h **(B2)**].

Scanning electron microscope (SEM) and TEM visualized changes of ultrastructure treated by bacteriocin. *L. monocytogenes* (Figure 7A0) and *C. sakazakii* (Figure 7B0) without treatment showed plump cell profiles with integrated biofilm in SEM. By TEM, cytoplasmic substances of both untreated indicators uniformly distributed (Figures 8A0, B0). However, after treated by bacteriocin, great changes of ultrastructure for both indicators have taken place. For *L. monocytogenes*, treatment by bacteriocin BM1122 for 0.5 h caused distorted profiles, biofilm-destruction, and pore-formation (Figure 7A1). Prolonged exposure time deteriorated cellular damage, the deformation was aggravated and pores became holes (Figure 7A2). Deformation may be a result of cytoplasm concentrated around cytomembrane and rarefied in the center (Figure 8A1). The nonuniform distribution of cytoplasm substance intensified at 2 h (Figure 8A2). The falling biofilm dispersed beside cells (arrows in Figures 8A1, A2). The appearance of *C. sakazakii* by BM1122 was different from *L. monocytogenes*. More pores were formed, as well as deformation was milder (Figure 7B1). The number and size of pores increased with treatment time (Figure 7B2). The abundant pores could pave more passages for outflow of cytoplasmic substances. So, the attenuated cytoplasm was more uniform (Figure 8B1). With longer treatment time, cell envelope was broken and cytoplasm substance was observed outside cells (arrows in Figure 8B2). In addition, BM1122 had very high sequence identity with DNA-binding proteins and conserved structure of histone-like DNA-binding protein. Antimicrobial peptide APP has been reported to have inhibitory activity due to efficient cell-penetrating efficiency, significant physiological changes and strong DNA-binding affinity (Li et al., 2016). Antimicrobial activity of BM1122 may also partly derive from DNA-binding action like Antimicrobial peptide APP (Li et al., 2016) and MBP-1 (Sousa et al., 2016), which needs to be further verified.

## Discussion

The *L. crustorum* MN047 strain, a poorly studied species, was isolated from koumiss. Koumiss is traditional fermented mare's milk and used as functional food for medical purposes (Wang et al., 2008; Vimont et al., 2017). A bacteriocin gene cluster was identified on the chromosome of *L. crustorum* MN047. In addition, multiple bacteriocin immunity proteins dispersed on chromosome far from the bacteriocin gene cluster. Moreover, multiple components of antimicrobial peptide transport system were found for *L. crustorum* MN047. These indicated that the *L. crustorum* MN047 might be able to produce multiple bacteriocins or antimicrobial peptides as our previous study on this strain (Yi et al., 2016). However, no exact bacteriocin encoding genes were found after identification in databases of BAGEL3 and antiSMASH. Therefore, bacteriocins produced by the *L. crustorum* MN047 were novel. After further investigation by combining complete genome and peptidome information, encouragingly eight novel bacteriocins produced by *L. crustorum* MN047 were identified. The function of eight novel bacteriocins was verified by cloning and heterologous expression. It proved that LC-MS/MS-based peptidome analysis combining complete genome is an efficient way to discover new bacteriocins.

Among the 8 novel bacteriocins, the BM1122 was originally annotated as “DNA-binding protein” in the NR database. The remarkable activity as bacteriocin in this study renovated its function annotation. The BM1029 had λ repressor-likeDNA-binding domains and the BMP11 had transmembrane region by InterPro analysis. The BM1556 was one of the putative bacteriocin structural genes in bacteriocin gene cluster. The other 4 novel bacteriocins were all originally annotated as “hypothetical protein.” Therefore, it is the first time to give a definite functional illustration for them. Biosynthesis mechanisms of other 7 bacteriocins in *L. crustorum* MN047 were mysterious. These bacteriocins might share the gene cluster of BM1556 because the core genes of biosynthesis and transport were flanked by four insertion sequence (IS) elements (Quintiliani and Courvalin, 1996). This composite transposon was mobile genetic element, which would facilitate the intracellular movement of carried genes (Hochhut et al., 2001). Namely, the composite transposon could play a key role in regulating multiple bacteriocins biosynthesis. As broad-spectrum antimicrobial activity, although these peptides were expressed in *E. coli*, the self-toxicity had prevented the production of bacteriocin beyond a limit.

**Figure 9:**
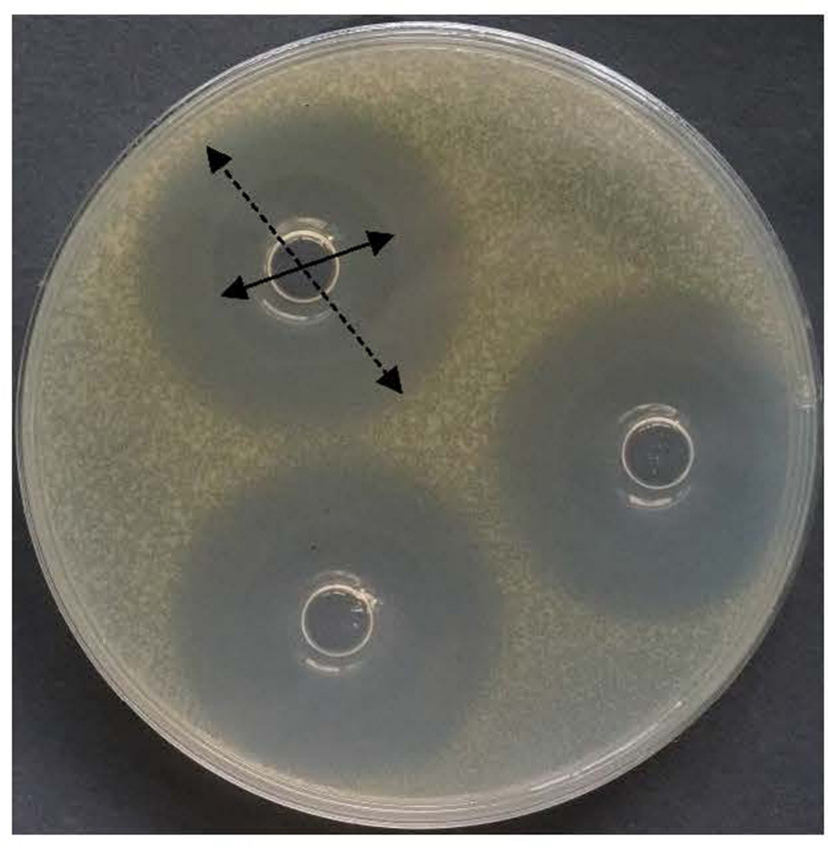
Inhibitory zone of bacteriocin BM1122 against antibiotic-resistant *Sakazakii*. Solid arrow is totally killed by the bacteriocin, dotted arrow is partially killed by the bacteriocin.

Nisin is the only widely used bacteriocin as biopreservative. However, it has a weakness of feeble activity to Gram-negative bacteria. Because of drug efflux pump mechanism of Gram-negative bacteria, it is a common phenomenon for many antimicrobials. Excitingly, the 10 peptides all showed broad-spectrum activity against not only Gram-positive but also Gram-negative bacteria. Both *Salmonella* and *C. sakazakii* were Gram-negative bacteria, and their multidrug-resistant strains were studied in this work. As shown in Figure 9, an obvious partial killing zone (dotted arrow) was found for Gram-negative pathogens. All data used in this study were total killing zone (solid arrow in Figure 9). Except for antibiotic-resistant *Salmonella* 557D, other resistant *Salmonella* strains were all resistant to the ten peptides. However, the resistance-spectrum of 557D contained all detected resistance-spectrum of 1006D. Sensitivity of multidrug-resistant *C. sakazakii* strains to the ten peptides was the same as that of wild strain. These indicate that antimicrobial mechanism of these peptides may be different from these prevalent antibiotics used in this study.

According to the study of Xiao et al. (2012), the EC_50_ value of EP-20 to *Phytophthora capsici* was 3.14 mg/L, that of GP-19 against *Verticillium dahlia* was 17.54 mg/L. EP-20 was antifungal with weak antibacterial activity while GP-19 had anti-Gram-positive, anti-Gram-negative and antifungal activity (Xiao et al., 2012). It is in accordance with the order of inhibitory competence in this study. However, the inhibitory activity of EP-20 and GP-19 was far less than that of BM1122.

The bacteriocin BM1122 had bactericidal action mode, which was powerful in controlling foodborne pathogens. The source of probiotic lactic acid bacteria and proteinaceous nature of BM1122 endow the potential as food preservative. According to SEM and TEM, action mechanisms of BM1122 against Gram-positive and Gram-negative bacteria were different. Action mechanisms of bacteriocins include cell envelope-associated mechanisms (e.g., pore formation, targeting lipid II) and intracellular mechanisms (interfering with DNA, RNA, and protein metabolism) (Cotter et al., 2013). The transmembrane region of BMP11 (Table 3) analyzed by InterPro may contribute to its antibacterial activity. For intracellular mechanism, DNA binding also is one approach, such as BM1029 and BM1122 analyzed by InterPro. How much contribution of the DNA binding of BM1029 and BM1122 to antibacterial activity needs to be verified in future study. However, the BM1122 also induced pore-formation (dotted arrows in Figure 7) and permeabilization of membrane according to thinned cytoplasm substance (dotted arrows in Figure 8). The pore-formation and membrane permeabilization could not be the results of DNA-binding. The difference and diversity of action mode contribute to antibacterial activity against broad pathogens, especially antibiotic-resistant strains.

In conclusion, combination of complete genome and peptidome was an excellent method in discovery of novel bacteriocins which were overlooked by general genome annotation. By which, eight novel bacteriocins and two antimicrobial peptides were identified from probiotic *L. crustorum* MN047. It is much more efficient than traditional ways in identification of bacteriocins.

## Author contributions

LY and XL designed the experiments. LY and LL performed the experiments and analyzed the experimental data. LY wrote this paper. All authors read and approved the final manuscript.

### Conflict of interest statement

The authors declare that the research was conducted in the absence of any commercial or financial relationships that could be construed as a potential conflict of interest.
