# Supplementary material for: Efficient Exploitation of Multiple Novel Bacteriocins by Combination of Complete Genome and Peptidome
Source: Front Microbiol. 2018 Jul 13;9:1567. doi: 10.3389/fmicb.2018.01567 (PMC6053492; doi:10.3389/fmicb.2018.01567)
Supplement: Supplementary file 1 [file Image_1.pdf]

Fig. S1 Fragments of eight hypothetical bacteriocins and two nonribosomal antimicrobial peptides identified by LC-MS/MS.

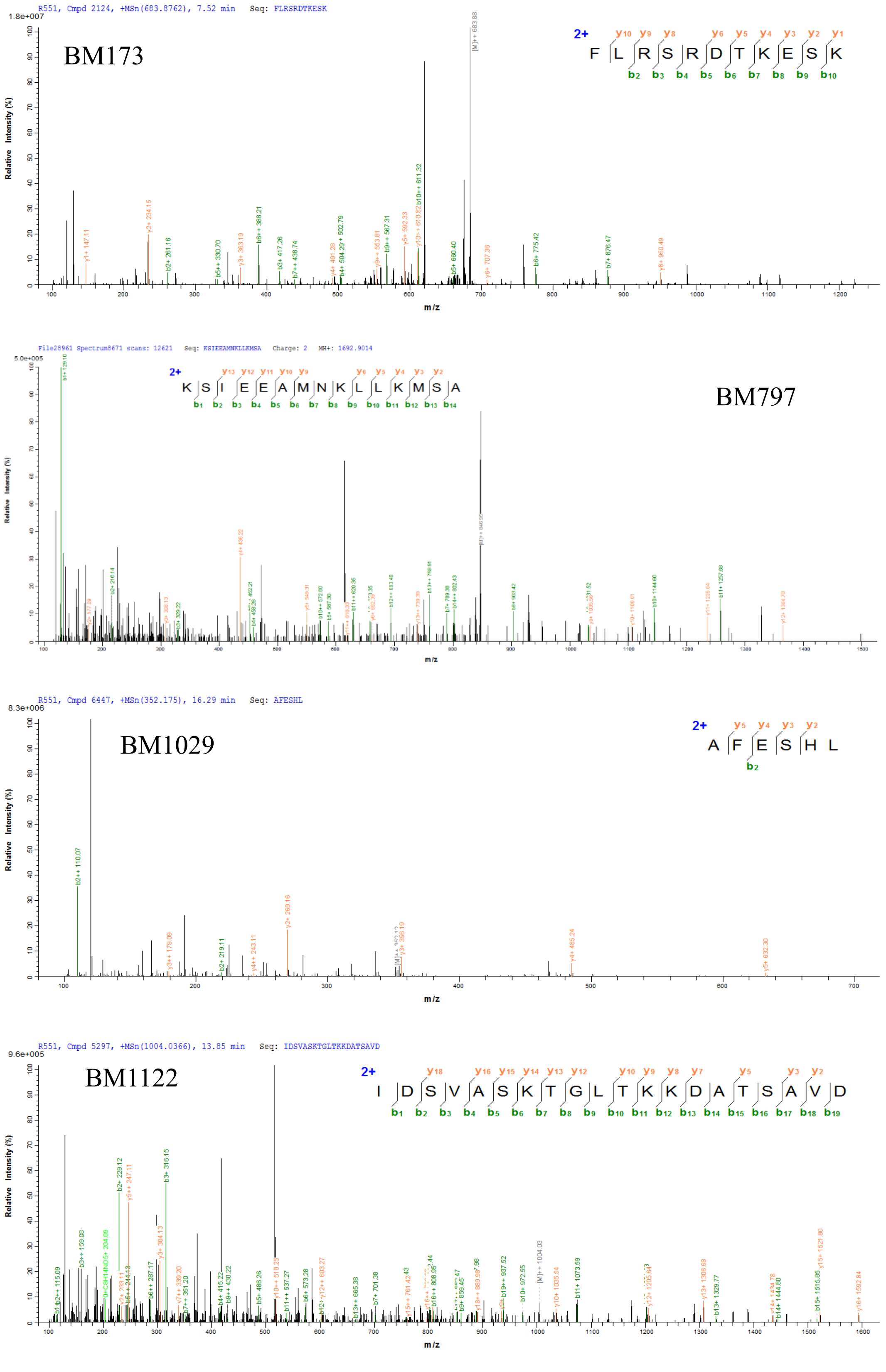

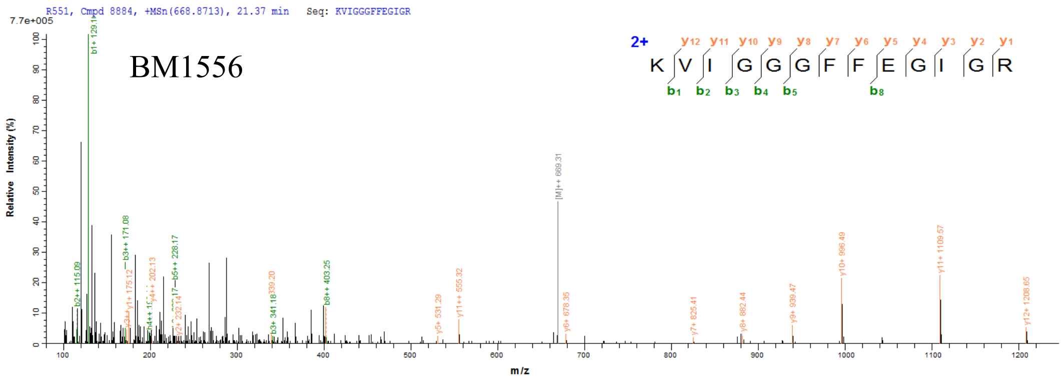
R551, Cmpd 8884, +MSn(668.8713), 21.37 min Seq: KVIGGGFFEGIGR
BM1556
2+
K V I G G G F F E G I G R
Relative Intensity (%)
m/z

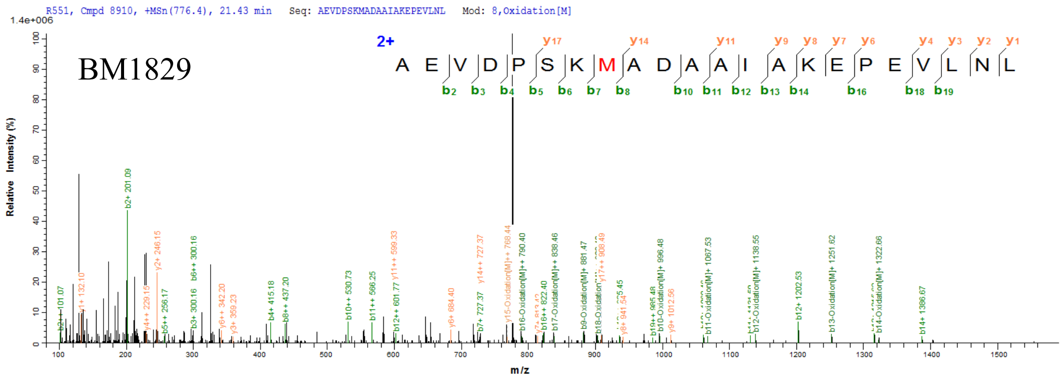
R551, Cmpd 8910, +MSn(776.4), 21.43 min Seq: AEVDPSKMADAAIAKEPEVLNL Mod: 8,Oxidation[M]
BM1829
2+
A E V D P S K M A D A A I A K E P E V L N L
Relative Intensity (%)
m/z

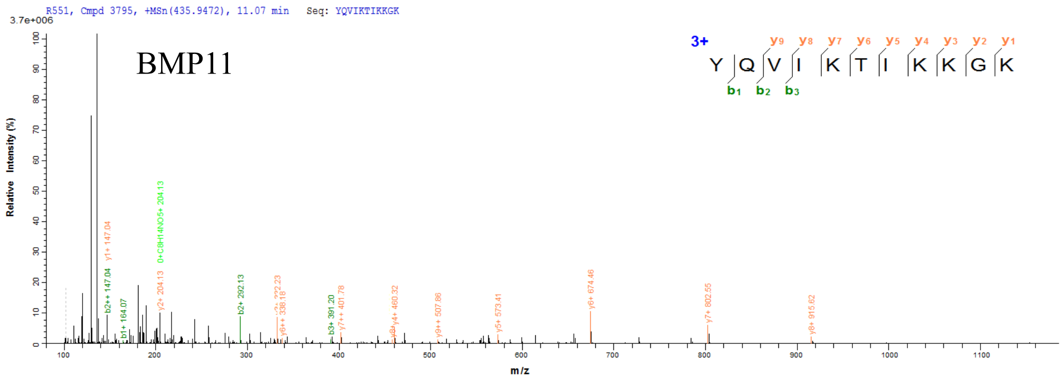
R551, Cmpd 3795, +MSn(435.9472), 11.07 min Seq: YQVIKTIKKGK
BMP11
3+
Y Q V I K T I K K G K
Relative Intensity (%)
m/z

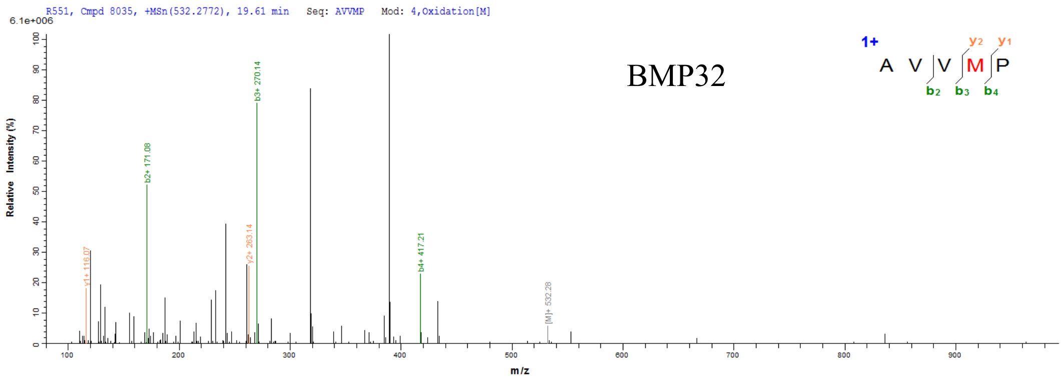
R551, Cmpd 8035, +MSn(532.2772), 19.61 min Seq: AVVMP Mod: 4,Oxidation[M]
BMP32
1+
A V V M P
Relative Intensity (%)
m/z

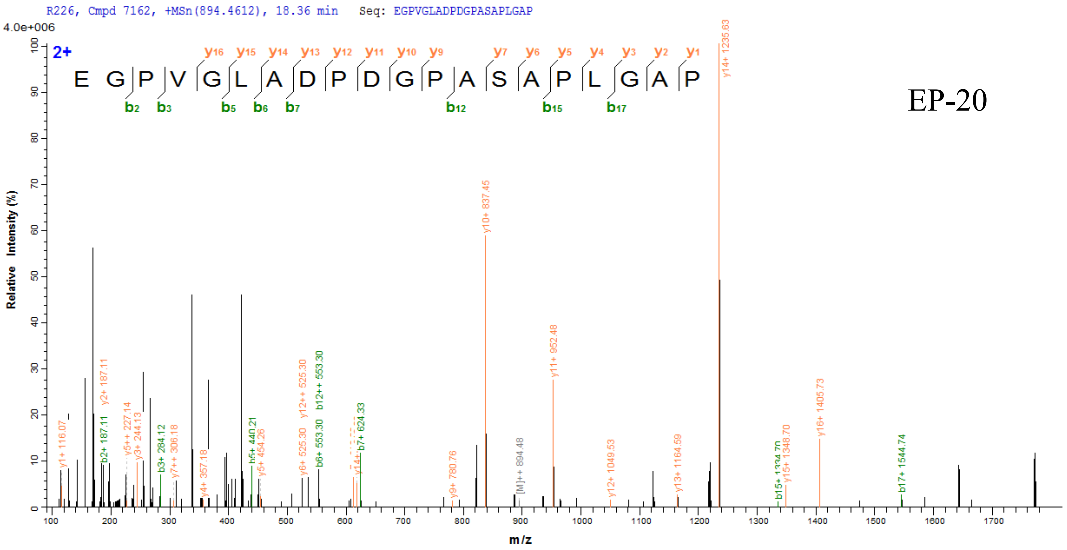
R226, Cmpd 7162, +MSn(894.4612), 18.36 min Seq: EGPVGLADPDGPASAPLGAP
4.0e+006
2+
E G P V G L A D P D G P A S A P L G A P
EP-20
Relative Intensity (%)
m/z

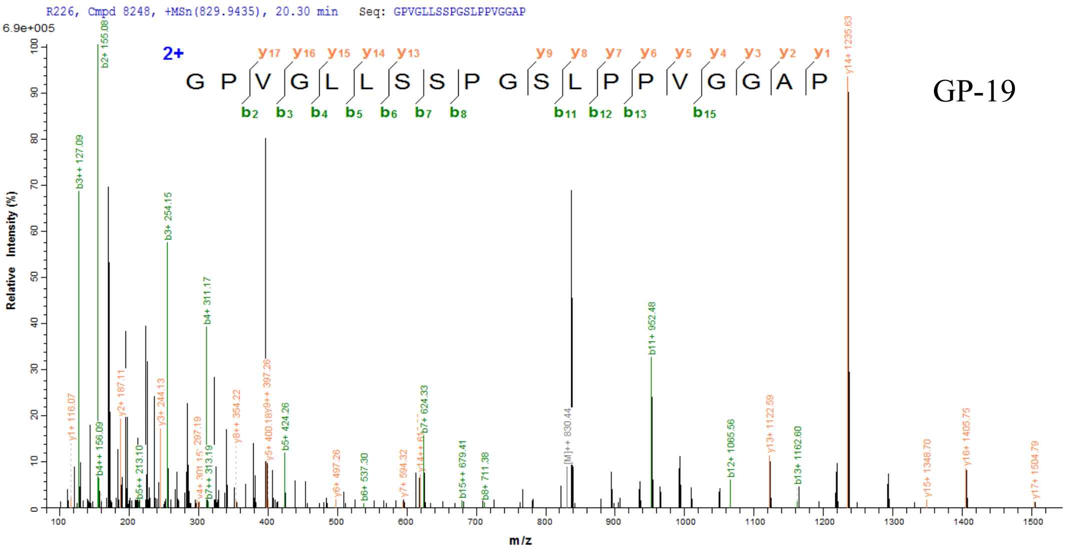
R226, Cmpd 8248, +MSn(829.9435), 20.30 min Seq: GPVGLLSSPGSLPPVGGAP
6.9e+005
2+
G P V G L L S S P G S L P P V G G A P
GP-19
Relative Intensity (%)
m/z
