# Supplementary figures and images for: Efficient Exploitation of Multiple Novel Bacteriocins by Combination of Complete Genome and Peptidome
Source: Front Microbiol. 2018 Jul 13;9:1567. doi: 10.3389/fmicb.2018.01567 (PMC6053492; doi:10.3389/fmicb.2018.01567)

**Fig. S2** Fragments of BM1122 identified by LC-MS/MS.

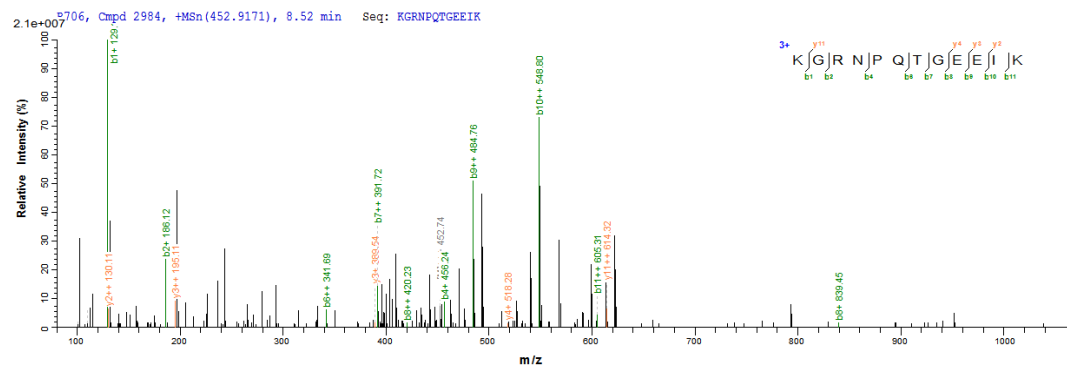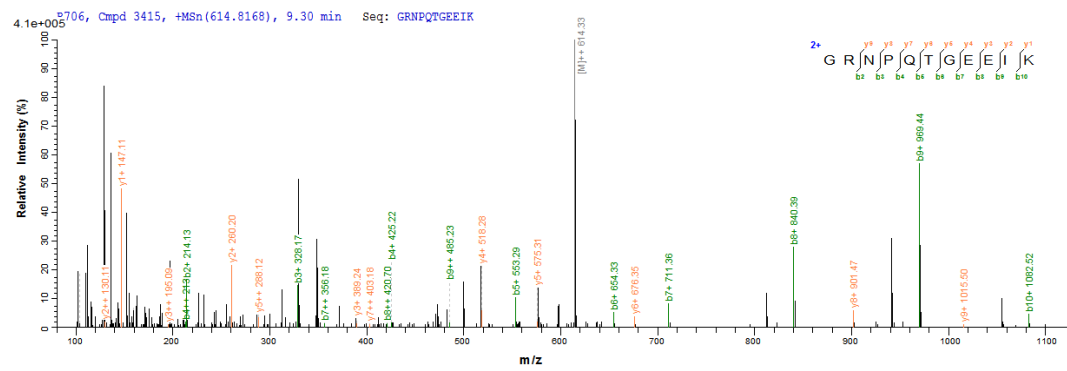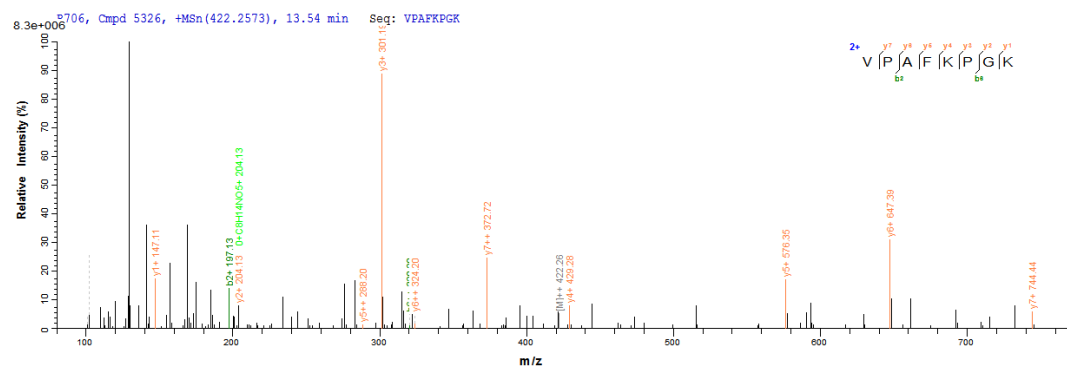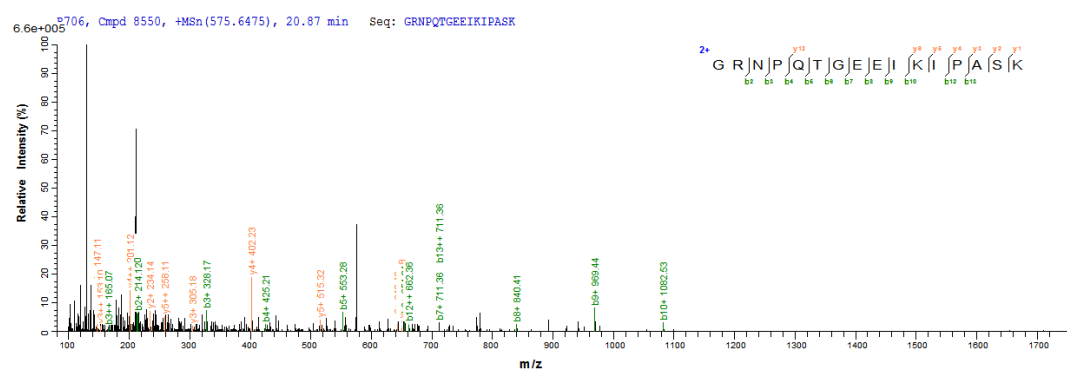

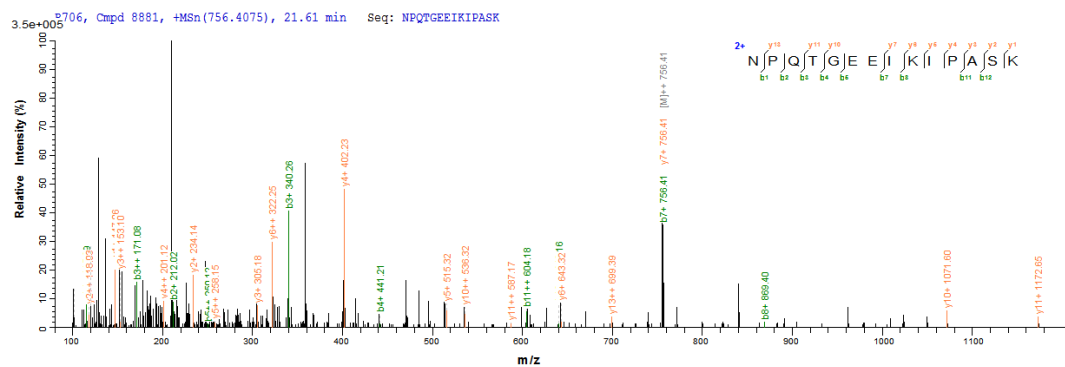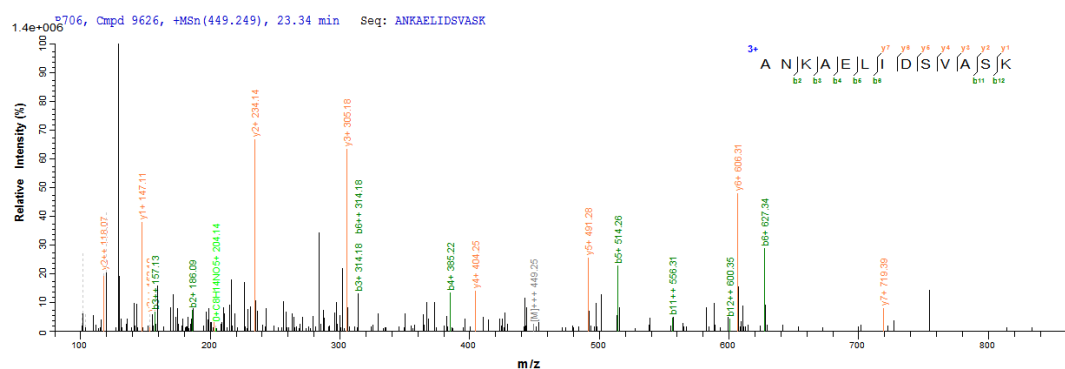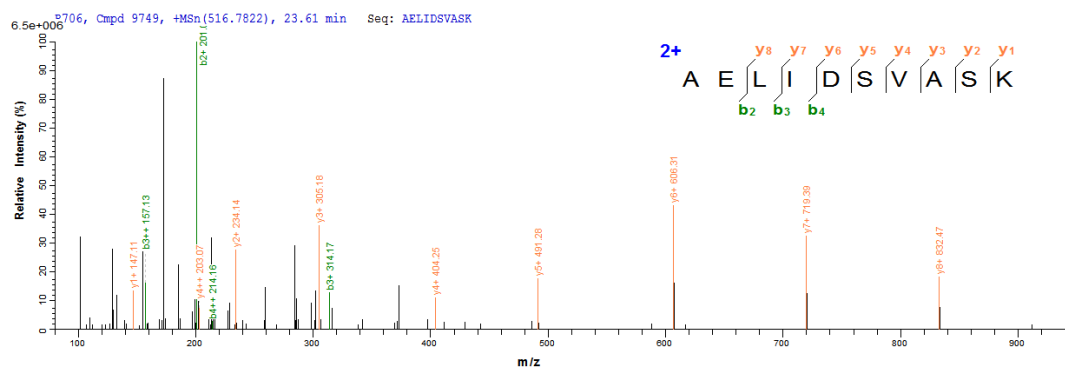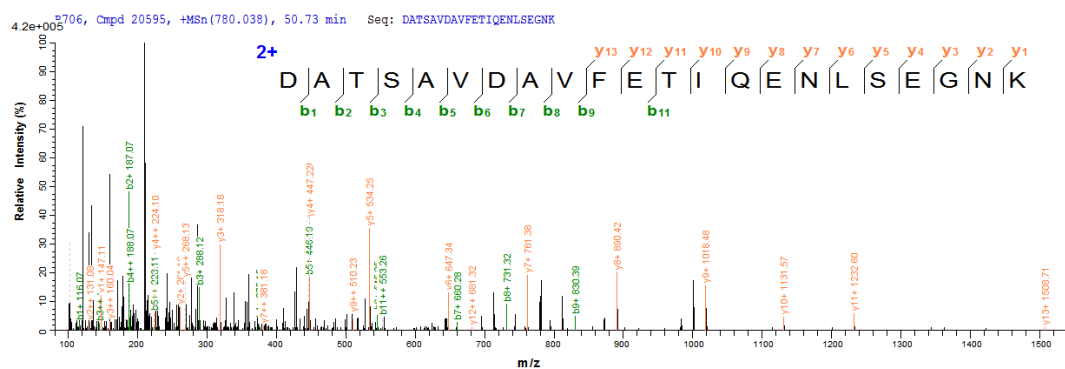

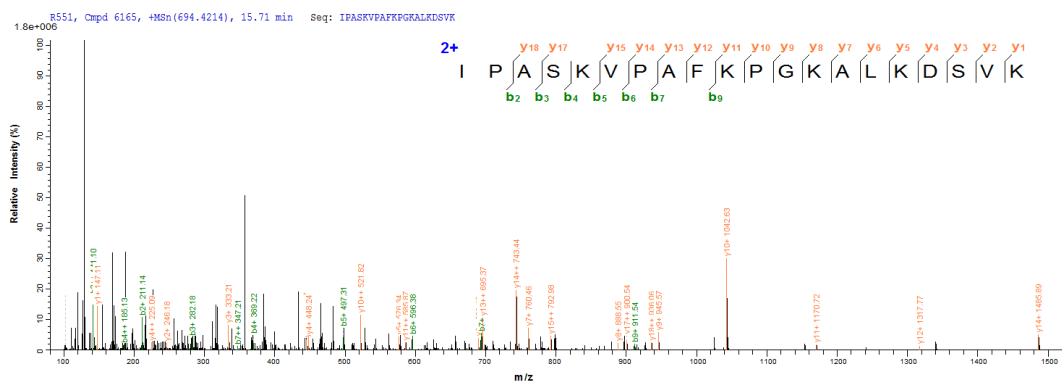

Supplement: Supplementary file 2 [file Image_2.pdf]
