# Supplementary material for: Efficient Exploitation of Multiple Novel Bacteriocins by Combination of Complete Genome and Peptidome
Source: Front Microbiol. 2018 Jul 13;9:1567. doi: 10.3389/fmicb.2018.01567 (PMC6053492; doi:10.3389/fmicb.2018.01567)
Supplement: Supplementary file 3 [file Table_1.docx]

**Table S1**

Concentration of kanamycin and IPTG optimized in this study and corresponding antimicrobial activity.

| Bacteriocin | Kanamycin (mg/mL) | IPTG (mM) | Antimicrobial activity^a^ (AU/mL) |
| --- | --- | --- | --- |
| BM173 | 10 | 0.4 | 1280 |
| BM797 | 8 | 0.7 | 640 |
| BM1029 | 10 | 0.4 | 1280 |
| BM1122 | 4 | 0.4 | 1280 |
| BM1556 | 7 | 0.6 | 640 |
| BM1829 | 5 | 0.4 | 640 |
| BMP11 | 4 | 0.4 | 1280 |
| BMP32 | 10 | 0.4 | 1280 |
| EP-20 | 10 | 0.2 | 320 |
| GP-19 | 8 | 0.4 | 640 |

a, *S. aureus* ATCC29213 was used as indicator.
